# Targeting the Essential Transcription Factor HP1043 of *Helicobacter pylori*: A Drug Repositioning Study

**DOI:** 10.3389/fmolb.2022.887564

**Published:** 2022-05-11

**Authors:** Federico Antoniciello, Davide Roncarati, Annamaria Zannoni, Elena Chiti, Vincenzo Scarlato, Federica Chiappori

**Affiliations:** ^1^ Department of Pharmacy and Biotechnology (FaBiT), University of Bologna, Bologna, Italy; ^2^ Istituto di Tecnologie Biomediche–Consiglio Nazionale Delle Ricerche (ITB-CNR), Segrate (Mi), Italy

**Keywords:** HP1043, *Helicobacter pylori*, antibiotic resistance, drug repositioning, essential transcription factor, virtual high-throughput screening (vHTS), MMGBSA

## Abstract

Antibiotic-resistant bacterial pathogens are a very challenging problem nowadays. *Helicobacter pylori* is one of the most widespread and successful human pathogens since it colonizes half of the world population causing chronic and atrophic gastritis, peptic ulcer, mucosa-associated lymphoid tissue-lymphoma, and even gastric adenocarcinoma. Moreover, it displays resistance to numerous antibiotics. One of the *H. pylori* pivotal transcription factors, HP1043, plays a fundamental role in regulating essential cellular processes. Like other bacterial transcription factors, HP1043 does not display a eukaryote homolog. These characteristics make HP1043 a promising candidate to develop novel antibacterial strategies. Drug repositioning is a relatively recent strategy employed in drug development; testing approved drugs on new targets considerably reduces the time and cost of this process. The combined computational and *in vitro* approach further reduces the number of compounds to be tested *in vivo*. Our aim was to identify a subset of known drugs able to prevent HP1043 binding to DNA promoters. This result was reached through evaluation by molecular docking the binding capacity of about 14,350 molecules on the HP1043 dimer in both conformations, bound and unbound to the DNA. Employing an ad hoc pipeline including MMGBSA molecular dynamics, a selection of seven drugs was obtained. These were tested *in vitro* by electrophoretic mobility shift assay to evaluate the HP1043–DNA interaction. Among these, three returned promising results showing an appreciable reduction of the DNA-binding activity of HP1043. Overall, we applied a computational methodology coupled with experimental validation of the results to screen a large number of known drugs on one of the *H. pylori* essential transcription factors. This methodology allowed a rapid reduction of the number of drugs to be tested, and the drug repositioning approach considerably reduced the drug design costs. Identified drugs do not belong to the same pharmaceutical category and, by computational analysis, bound different cavities, but all display a reduction of HP1043 binding activity on the DNA.

## 1 Introduction

*Helicobacter pylori* is one of the most widespread and successful human pathogens since it colonizes half of the world population (Hooi et al., 2017). Infected people carry this bacterium for decades or even for life; if untreated, *H. pylori* can remain clinically silent for a long time due to the dynamic equilibrium between the bacterium and the host or evolve into chronic gastritis or even more severe diseases such as atrophic gastritis, peptic ulcer, MALT-lymphoma, or gastric adenocarcinoma. Despite its declining incidence rate, gastric cancer remains the fifth most common malignancy in the world and the third leading cause of cancer-related death (Sugano et al., 2015). *H. pylori* infections are currently treated with a combination of a proton pump inhibitor and different antibiotics; unfortunately, the available therapies are losing efficacy because of the antibiotic resistance insurgence, with eradication cure rates lower than 70% (Vianna et al., 2016). For instance, due to a constant increase in *H. pylori* resistance to clarithromycin, the triple clarithromycin-based treatment has become progressively less efficacious (Jenks and Edwards, 2002). For this reason, the World Health Organization (WHO) has included *H. pylori* in a global priority list of 12 antibiotic-resistant bacterial pathogens to help the scientific research prioritize the discovery and development of new antibiotics (WHO, 2017). Alongside multidrug-resistant bacteria, antibiotic-resistant *H. pylori* strains pose a major public health issue, and novel antibacterial strategies against *H. pylori* persistent infection are overdue.

Bacterial pathogens sense the host environment and respond with the expression of gene products required to adapt to a particular niche. These adaptive responses rely on transcriptional regulatory circuits that control the coordinated expression of several proteins, including virulence factors, in space and time (Seshasayee et al., 2006). *H. pylori* makes no exception to this paradigm, despite a remarkable paucity of annotated transcriptional regulators. To date, only 17 transcriptional regulators have been identified and characterized to different extents (Danielli and Scarlato, 2010). Among them, the HP1043 regulator seems to play an essential role. The *hp1043* gene cannot be deleted, nor the amount of protein modulated, supporting the hypothesis that HP1043 could be involved in the regulation of fundamental cellular processes (Schär et al., 2005). To shed light on this possibility, by chromatin immunoprecipitation-sequencing (ChIP-seq), Pelliciari et al. (2017) comprehensively identified genome-wide the HP1043 *in vivo* binding sites.

**FIGURE 1:**
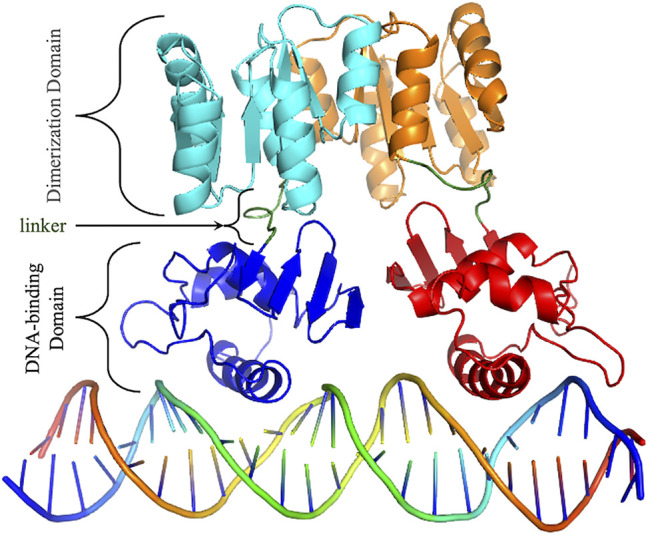
HP1043 structure. Cartoon representation of HP1043 bond to the DNA model. Protein domains and linker are evidenced.

HP1043 is a 223 amino acid long protein, composed by a dimerization domain (or response regulatory, residues 2–112) and a DNA-binding domain (OmpR/PhoB-type, residues 118–216) connected by a short 5 amino acid linker (residues 113–117) (Figure 1).

Intriguingly, the study showed that HP1043 dimerizes and binds *in vivo* the promoter of genes involved in all the fundamental processes of the bacterial life cycle, namely, DNA replication, RNA transcription and translation, and energy production and conversion (Pelliciari et al., 2017). The resulting head-to-head dimer conformation, different from the canonical head-to-tail of the OmpR/PhoB subfamily, was theoretically obtained, based on experimental mutagenesis data and inter-domain linker flexibility evaluation (Zannoni et al., 2021). HP1043 appears to be fundamental for the fitness of the bacterium, a prerequisite for a successful infection, and it is a pivotal regulator on which *H. pylori* relies to modulate its metabolism and growing behavior. For these reasons, HP1043 makes a promising candidate to develop novel antibacterial strategies. Essential transcriptional regulators are appealing targets for the development of new antibiotics (González et al., 2018). Indeed, the inhibition of such regulators leads to the altered expression of crucial genes for cell viability. These regulators have no counterpart in humans while being specific for a particular microorganism. They are usually small soluble proteins that are easy to purify (Roncarati et al., 2022). In particular, the ease of handling makes the transcriptional regulators appropriate for experimental approaches such as *in vitro* binding assay, inhibition tests, and co-structural analysis. Moreover, they are ideal proteins for *in silico* approaches, such as structure-based virtual screening for compounds that are able to bind and hinder the regulatory function. The recombinant purified HP1043 of the *H. pylori* 26695 strain has been used to set up a fluorescence-based thermal shift assay to identify HP1043 binding molecules. This approach has been used to carry out a high-throughput screening of 1120 small molecules FDA-approved for human use and off-patent which resulted in the identification of 14 compounds that bind to the native state of HP1043. Notably, seven identified HP1043 binders were natural flavonoids interacting with the helix-turn-helix DNA-binding motif. These natural compounds inhibited the *in vitro* DNA-binding activity of HP1043, and four of them showed bactericidal activity against *H. pylori* (González et al., 2019b). The same screening led to the identification of other FDA-approved drugs that can form stable complexes with HP1043, including the 1,4-dihydropyridine calcium channel blocker nicardipine (González et al., 2019a), opening the possibility of a drug-repurposing approach for the treatment of *H. pylori* infection.

An alternative approach in the field of drug discovery is based on the generation of high-resolution structural data of the selected target followed by a repositioning strategy (Pushpakom et al., 2019) that might potentially hinder the target’s function. To this aim, our group has recently combined biochemical and computational approaches to characterize the binding architecture of the HP1043 regulator of the *H. pylori* G27 strain to a selected target promoter, P_*hp1227*_. Experimental data combined with the available HP1043 NMR structure were used as restraints to guide an in silico protein–DNA docking. The generated model shows an HP1043 dimer interacting in a head-to-head conformation with both the major and minor grooves of a target DNA sequence (Zannoni et al., 2021). Moreover, the dimer conformation is known to increase the binding to the promoter region on DNA (Simonovic and Volz, 2001). In this study, we screened a collection of about 14,350 small molecules, composed of approved drugs (FDA approved, NIH clinical collection, Drug Bank approved, and Therapeutics Target Database approved) and “substances with the main target as transcription factors” included in the ZINC15 database. Virtual high-throughput screening (VHTS) was performed on both complexes, HP1043 dimer free, to identify molecules that can bind to the protein–DNA interface, and HP1043 dimer bound to DNA, to identify molecules that can bind the complex and potentially induce DNA unbinding. Promising complexes were analyzed by molecular dynamics as obtained by docking to evaluate the drug-binding effect dynamically. This work aimed to evaluate the reposition of approved drugs and transcription factors known as ligands to the HP1043 *H. pylori* transcription factor, selecting by computational methods a subset of molecules able to prevent DNA binding. Selected drugs were afterward experimentally tested; and among these, three returned promising results showing, *in vitro*, an appreciable reduction of the DNA-binding activity of HP1043.

## 2 Materials and Methods

### 2.1 Molecular Docking

The previously obtained molecular models of dimeric HP1043 alone and bound to DNA were employed (Zannoni et al., 2021). HP1043 model dimer bound to the DNA was obtained by a data-driven docking, employing NMR data, and domain X-ray deposited data, and the residues involved in DNA binding were experimentally validated (for details see (Zannoni et al., 2021)). Both complexes were relaxed through 100 ns of molecular dynamic simulation and the most representative conformations obtained from cluster analysis were employed for the virtual screening step.

We used the ZINC database (http://zinc15.docking.org/) (Sterling and Irwin, 2015) to select a ligand dataset of about 14,350 molecules from five different datasets. In detail, we selected substances that can target a transcription factor (TF), Drug Bank-approved molecules (DBa), Food and Drug Administration-approved drugs (FDAa), Therapeutic Target Database-approved drugs (TTDa), and NIH clinical collection (Ncc).

Docking simulations were performed using Autodock 4.2 and AutoDockTool (ADT) interface (Morris et al., 2009). An in-house Perl script pipeline was employed for ligand preparation, parameter file for docking simulation, Slurm job-scheduling input file, and docking results analysis, while the protein structure was prepared with the ADT interface, a docking box of 126 × 126 × 126 points, with a spacing of 0.375 Å, was centered on the protein–DNA complex, including almost the whole complex, excluding the DNA underside. Considering the unbound complex, two docking boxes were prepared with the same characteristics as the previous one, both centered on the HP1043 DNA-binding domain of each protein chain, since a single box cannot include the entire docking surface.

The affinity maps for all the atom types available in AutoGrid were pre-calculated. Docking simulations were performed by treating the protein as rigid, ligands as flexible, and 50 runs for each simulation of the Lamarckian genetic algorithm were performed using the AutoDock 4.2.6 suite (Morris et al., 2009). Selected molecules were subjected to a deep re-docking screening of 200 runs per simulation, maintaining the other conditions.

The pipeline parses the docking results to identify, for each simulation, the most representative conformation, that is, the best energy conformation and all the conformations with a docking energy within 1 kcal/mol from the first ranked solution. Docking energy, cluster population, estimated Ki value, and atomic coordinates of each selected solution are extracted. The pipeline reconstructs the coordinate files of protein–ligand complexes. Moreover, final molecules were selected based on the Ki value, in the order of size of pM or lower and only the re-docking results, on cluster numerosity, greater than 20 units.

### 2.2 Molecular Dynamics

The molecular dynamics of the HP1043 bound or unbound to DNA molecule, as obtained by molecular docking, and in complex with a ligand selection was performed using AMBER 18 (Salomon-Ferrer et al., 2013). To parameterize the complex, ff14SB (Maier et al., 2015) was employed for the protein, OL15 (Cheatham and Case, 2013) force field for DNA, and water was treated as an optimal point charge. The total charge of each complex was balanced with Na^+^ counter ions, and the solution molarity was set to 150 mM adding Na^+^ and Cl^−^ ions. Solvated complexes were minimized for 1,000 steps, and heated until 300 K in 100 ps followed by 50 ps of NPT equilibration. Ten simulations of 10 ns each were performed using the molecular mechanics generalized Born surface area (MMGBSA) protocol, employing periodic boundary conditions. Trajectory and energetic analyses were performed using the cpptraj and MMBPSA.py tools (Miller et al., 2012). In detail, Cα root mean square deviation (RMSD), per-residue root mean square fluctuation (RMSF), the distance between protein interface residues and specific DNA nucleotides, and Cα hierarchical agglomerative clustering analysis were evaluated using cpptraj. To perform binding energy analysis on protein–ligand and protein (ligand)–DNA complexes, the tool is MMPBSA.py was applied on 50 equally distributed frames along the joint trajectory, and the solvation free energy was evaluated using the modified generalized Born (GB) model (Onufriev et al., 2004) using 1.0, 0.8, and 4.85 values for α, β, and γ, respectively, and ions concentration was set at 0.150 M.

LigPlot+ (Laskowski, 2011) was employed for the identification of interacting residues and its classification as contacts or residues establishing hydrogen bonds.

### 2.3 Chemicals

Plerixafor and ribociclib were purchased from AdooQ^®^ Biosciences (United States), and oxcarbazepine and temoporfin were purchased from Cayman Chemical (United States), while dihydroergotoxine, tetraethylenepentamine, and trientine were purchased from Sigma-Aldrich (United States). All drugs were properly stored at −20°C according to the manufacturer’s indications. For each compound, stock solutions of 16.67 mM were prepared by dissolving the powder in either H_2_O or 100% DMSO. The pH of aqueous solutions was adjusted when needed.

### 2.4 Overexpression and Purification of Recombinant His6-HP1043

Recombinant N-terminal His-tagged HP1043 wild-type and mutant proteins were overexpressed in *Escherichia coli* BL21 DE3 cells transformed with plasmid pTrc::1043 (Supplementary Table S1). For electrophoretic mobility shift assays (EMSA), HP1043 was purified as previously described (Zannoni et al., 2021). Briefly, the cells were incubated with lysis buffer (50 mM Tris-HCl pH 8.0, 500 mM NaCl, and 10 mM imidazole), lysozyme (0.5 mg/ml), and 1× cOmplete™ Protease Inhibitor Cocktail (Roche, Basel, Switzerland) for 1 h at 4°C. Afterward, the mix was sonicated and centrifuged at 22,000 × *g* for 30 min at 4°C. The supernatant was collected and incubated in a batch with Ni^2+^−NTA resin for His-tagged purification. The bound protein was eluted using an imidazole gradient (10–250 mM) and dialyzed twice against the store buffer (50 mM Tris-HCl pH 8, 300 mM NaCl, and 10% glycerol). Protein purity was assessed by SDS-PAGE and its concentration was determined by measuring the absorbance at 280 nm using a NanoDrop^®^ spectrophotometer (Thermo Fisher Scientific, Waltham, MA, United States).

### 2.5 Electrophoretic Mobility Shift Assays

The DNA-binding activity of the recombinant HP1043 in the presence of putative inhibitors was assessed by EMSAs as already described (Zannoni et al., 2021). In brief, a 190-base pair (bp) promoter region of *hp1227* (strain 26695 annotation; P_*hp1227*_) (Tomb et al., 1997) including the HP1043 binding consensus sequence was amplified from pGEMt-P1227_WT (Supplementary Table S1) by PCR with oligos Php1227_EMSA_F and Php1227_EMSA_R and used as a target sequence of HP1043 (Supplementary Table S2). A 127-bp sequence obtained through PCR amplification with oligos 16S_RTF and 16S_RTR was used as a non-specific control. The recombinant HP1043 protein (4 µM) was mixed with approximately 10 ng of target promoter probe and 10 ng of non-specific DNA probe in a 25 µl reaction volume containing 1 × binding buffer (10 mM Tris-HCl pH 7.5, 40 mM KCl, 100 μg/ml BSA, 1 mM DTT, and 5% glycerol). For binding interference assays, putative binders were added to final concentrations of 1, 0.5, 0.2, 0.1 and 0.05 mM to the mixtures of protein and DNA. Single ready-to-use aliquots were thawed and immediately diluted to the desired concentration and thereby use the same volume of ligand solution in EMSA analysis. Binding assays with DMSO (6% (v/v)) or H_2_O instead of inhibitors and 1 × binding buffer instead of the protein were included as controls. The reactions were incubated at room temperature for 30 min and then separated on a 6% non-denaturing polyacrylamide gel 0.5 × running buffer (45 mM Tris pH 7.5 and 45 mM orthoboric acid) at 90 V for 75 min, using a Mini Gel Tank apparatus (Invitrogen, Waltham, MA, United States). DNA bands were stained with 1 × ethidium bromide and visualized using a Gel Doc XR+ image analyzer (BioRad, Hercules, CA, United States).

## 3 Results

### 3.1 Repositioning VHTS

#### 3.1.1 HP1043–DNA Complex

To identify the possible ligands of the HP1043–DNA complex, a dataset of 14,350 approved molecules was screened. The first docking screening returned 180 ligands with a Ki value in the order of magnitude of pM. These were subjected to a re-docking procedure to identify a subset of the best candidates. The re-docking simulation returned 323 conformations (multiple poses of the same ligand were allowed) with a Ki value in the pM order of magnitude. Among these, 50 conformations corresponding to 41 different ligands displayed cluster numerosity greater than 20 units, for these reasons, were considered interesting candidates.

**FIGURE 2:**
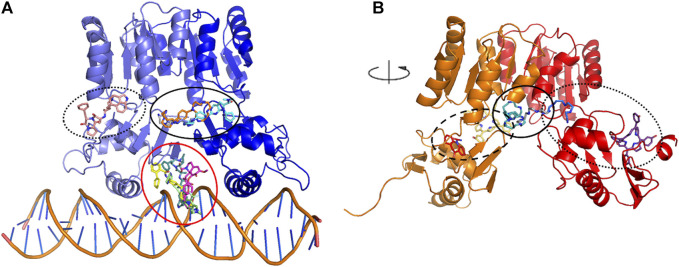
Docking binding sites for HP1043–DNA **(A)** and HP1043 **(B)**. Main binding sites are evidenced in circles, interface between the dimerization and the DNA-binding domains, and/or between the two HP1043 chains in solid line, A chain in dashed line, B chain on dotted line, and interface between protein and DNA in red line.

A subset of eight ligands bound at the interface between the dimerization and the DNA-binding domains, and/or between the two HP1043 chains and one docked at the B chain external surface, the remaining ligands bound at the interface between the protein and DNA (Figure 2A). The latter can be considered ligands with lower specificity for the free form of HP1043; instead, the first eight ligands can be regarded as promising candidates for targeting HP1043, also in free form.

#### 3.1.2 HP1043 Complex

We applied the aforementioned protocol to the HP1043 dimer unbound to DNA to identify molecules able to bind the transcription factor before binding to the DNA. The first docking screening returned 155 ligands, corresponding to 161 different poses, with an estimated Ki in the fM and pM order of magnitude and sufficiently represented (see Materials and Methods for details). These were re-docked as previously described, and only two ligands in six different conformations satisfied the Ki and representative restraints. Here, we also considered ligands with the nM Ki value with cluster numerosity greater than 100 units, returning overall 19 conformations, corresponding to 15 different ligands or same ligands but localized in different binding sites. Among these, seven localized at the interface between the two chains, one bound the DNA-binding domain of the A chain at the internal surface. In contrast, nine localized at the external surface, two at the interface between the two domains of the B chain, and the last one bound to the external surface of the B chain (Figure 2B).

To compare docking results with the literature, available data (González et al., 2019a; González et al., 2019b) were analyzed with our docking protocol. Different from the literature, we docked the small molecules to HP1043 dimeric conformation; both in the DNA bound and unbound form. Even so, the obtained computational binding energy was comparable to the published results (Supplementary Table S3 compared to docking binding energy in (González et al., 2019a; 2019b)). Binding energies ranged from −5.03 to −10.07 kcal/mol for the DNA-bound complex and from −5.83 to −10.10 kcal/mol for the unbound complex. Therefore, we selected our screening molecules with a computational binding energy lower than about 10 kcal/mol.

### 3.2 Dynamical Evaluation of Selected Drugs

**FIGURE 3:**
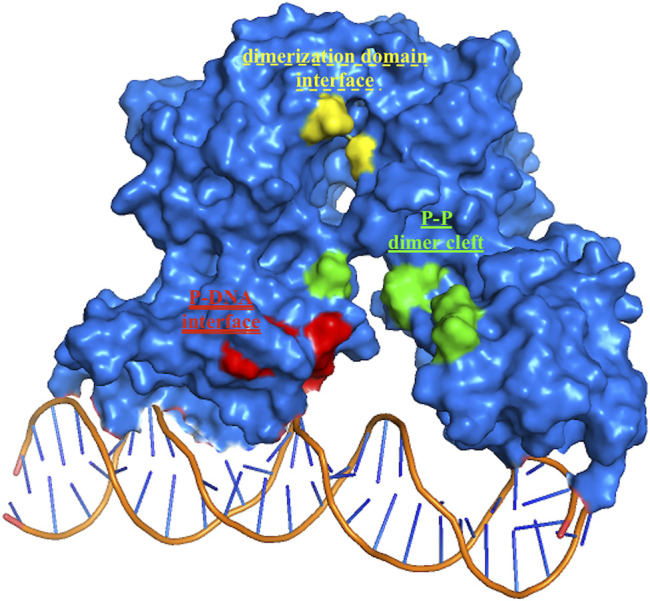
HP1043 binding sites. Residues belonging to the three principal binding sites, in green residues at the dimer cleft (P–P and single underlined in Table 2), in yellow residues at the dimerization domains interface (dotted-underlined in Table 2), and in red residues at the A chain–DNA interface (double-underlined in Table 2).

Among the 56 docking results selected with VHTS (listed in Supplementary Table S4), “Not for sale” or too expensive compounds and ligands bound only to DNA molecule were excluded. A subset of 13 compounds (Table 1) corresponding to 17 different binding poses was submitted to MMGBSA molecular dynamics simulation to obtain an accurate evaluation of binding mode and energy (Table 2) in the same complex conformation as docking results. Otherwise, the first 11 complexes listed in Table 2 (HP1043–DNA) were simulated and bound to the HP1043 dimer in the complex with the DNA molecule, and the last six complexes listed in Table 2 (HP1043) were simulated and bound to the HP1043 dimer free form. The selected molecules are mainly representative of the different binding sites. One at the dimer cleft (in green in Figure 3, P–P in Table 2), involving the residues of both chains (single underlined in Table 2), one at the dimerization domain interface between the two chains (in yellow in Figure 3, dotted-underlined in Table 2) and one with a lower specificity at the protein–DNA interface (in red in Figure 3, P-DNA in Table 2), which involves, in addition to the residues of the A chain, the DNA molecule (double-underlined in Table 2). Three other binding sites were identified. One localized at the domain interface of the B chain (Bi in Table 2) and partially involved residues of the dimer cleft, one localized on the external surface of the A chain (A ext in Table 2) and the other on the external surface of the B chain (B ext in Table 2).

**TABLE 1:** Selected drugs.

Ligand	Molecule name	Molecule sketch	Ligand	Molecule name	Molecule sketch
ZINC000012503187 (FDAa, DBa, and TTDa)	conivaptan	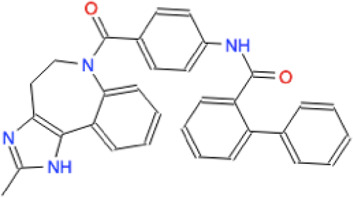	ZINC000036701290 (FDAa and DBa)	ponatinib	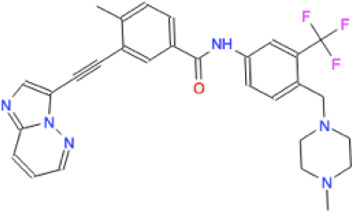
ZINC000014880002 (TTDa)	dihydroergotoxine	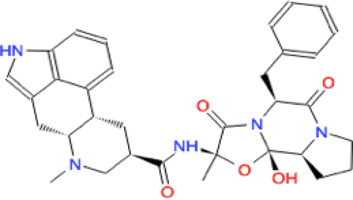	ZINC000072316335 (FDAa and DBa)	ribociclib	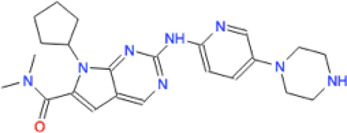
ZINC000052955754 (FDAa, DBa, and TTDa)	ergotamine	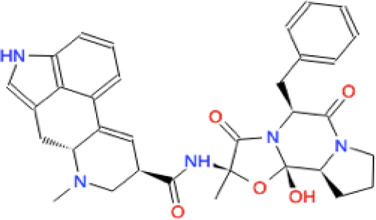	ZINC000003934128 (DBa)	temoporfin	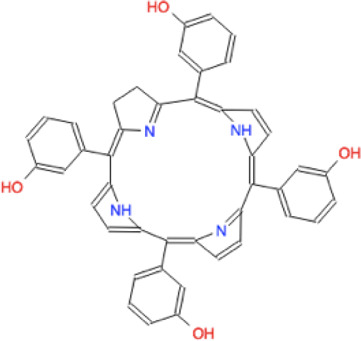
ZINC000001566899 (DBa and TTDa)	hexafluronium	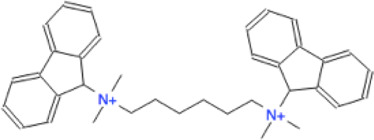	ZINC000019363,537 (FDAa and DBa)	tetraethylenepentamine	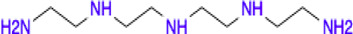
ZINC000098023177 (FDAa and Dba)	osimertinib	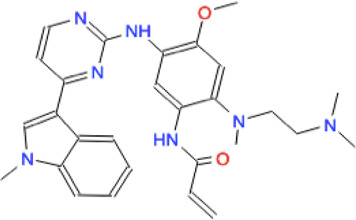	ZINC000019364225 (FDAa, Dba, and TTDa)	trientine	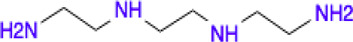
ZINC000000004724 (FDAa, DBa, and TTDa)	oxcarbazepine	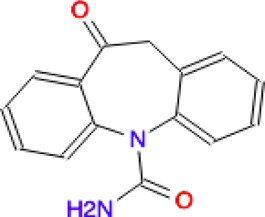	ZINC000003978083 (DBa and TTDa)	tubocurarin	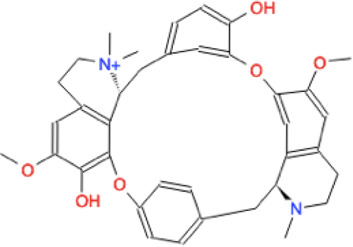
ZINC000022443609 (FDAa, DBa, and TTDa)	plerixafor	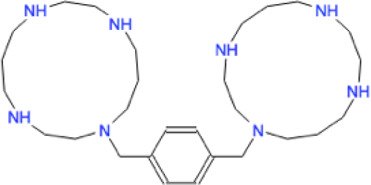	-		

FDAa = Food and Drug Administration approved; DBa = Drug Bank approved; TTDa = Therapeutics Target Database approved

**TABLE 2:** HP1043 drug docking and MMGBSA.

	Binding Site	Molecule name	Binding energy (kcal/mol)	# cluster	Ki	Docking interaction residues (LIGPLOT)	MMGBSA P-L (kcal/mol) (SD)	MD interaction residues (Ligplot)
HP1043-DNA	P-DNA	hexafluronium	−15.41	40	5.06 pM	Contacts: Y360, V365, F372, K417, M418, P421, DNA	−20.1 (±6.93)	Contacts: Y360, R363, E364, V365, K417, DNA
		ponatinib	−13.08	26	256.40 pM	Contacts: Y360, F372, DNA	−35.27 (±12.22)	Contacts: DNA
						Hbonds: DNA		
		osimertinib	−12.89	22	356.10 pM	Contacts: DNA	−10.56 (±4.78)	Contacts: DNA
						Hbonds: DNA		
		tubocurarin	−12.46	51	737.90 pM	Contacts: P148, F149, V365, DNA	−20.99 (±8.11)	Contacts: DNA
						Hbonds: K194, DNA		
		ergotamine	−12.46	26	736.10 pM	Contacts: Y360, V365, E366, V367, F372, DNA	−20.62 (±5.61)	Contacts: L126, I135, Y137, DNA
						Hbonds: DNA		Hbonds: DNA
		conivaptan	−12.42	35	791.50 pM	Contacts: I358, V365, M418, P421, DNA	−34.95 (±6.76)	contacts: L349, Y360, R363, V365, L375, I414, K417, M418, P421, L422, DNA
						Hbonds: Y360, DNA		Hbonds: DNA
	P–P	**plerixafor**	−23.84	54	3.37 aM	Contacts: R114, E150, S290, K357, I359	−75.23 (±11.93)	Contacts: E174, S290, E364
						Hbonds: E133, K145, E355, E364		Hbonds: D131, E133, D354, E355
		**tetraethylenepentamine**	−15.57	34	3.86 pM	Contacts: S291, S352	−52.48 (±12.59)	Hbonds: W173, E174, E175, P176, E177 (ligand is leaving the complex)
						Hbonds: E133, K145, E355, E364		
		**trientine**	−13.84	51	71.87 pM	Contacts: E133, K145, E364	−44.38 (±12.42)	Contacts: E133
						Hbonds: E355		Hbonds: D131, E132, E355, E364
		**ribociclib**	−12.7	25	487.80 pM	Contacts: R114, F115, P130, K145, K147, T153, H154, R157	−24.23 (±5.23)	Contacts: A111, L113, F115, P130, F149, E150, T153
						Hbonds: W116, E133, E150, E364		Hbonds: E110, R114
	B ext	**dihydroergotoxine**	−12.39	52	827.20 pM	Contacts: D264, I265, K288, Y439, K441, P442, A443, E446	−6.65 (±9.33)	Contacts: M263, D264, I265, R266, N267, K288 (ligand is leaving the complex)
						Hbonds: H289, E445		
HP1043	P–P	**plerixafor**	−17.65	35	116.01 fM	Contacts: K286, F310, Q312, G313	−84.34 (±18.64)	Contacts: A111, L113, E174, P176, K286, T350, S352
						Hbonds: E175, E287, E311, A314, D315, D354		Hbonds: E175, E177, E287, S290, D354
		**tetraethylenepentamine**	−12.86	20	373.37 pM	Hbonds: E174, E175, E311	-58.09 (±13.58)	Contacts: A111
								Hbonds: E174, E175, E287, E311
	Bi	**oxcarbazepine**	−9.94	103	51.39 nM	Contacts: S341, V343, I344, I351, L375, T376, A379, R382	−22.92 (±3.00)	Contacts: V343, I344, I351, I358, L375, T376, A379, R380
						Hbonds: N342, R380		
		**temoporfin**	−12.23	146	1.09 nM	Contacts: E333, L336, F338, W339, N342, P353, E356, V367, T376, H381	−29.49 (±5.45)	Contacts: D92, M224, L336, F338, W339, P353, E356, T376
						Hbonds: R337, K368		Hbonds: R337
	A ext	**tetraethylenepentamine**	−13.68	21	94.13 pM	Hbonds: D170, W173, E174, E175	−49.66 (±12.8)	Hbonds: E174, E175, E311
		**temoporfin**	−11.18	102	6.41 nM	Contacts: I121, E122, G124, D160, Q161, I201, T203, F214, Y216, P217, C221	−36.01 (±5.20)	Contacts: I121, E122, I123, G124, Q161, I162, M195, L199, I201, S202, T203, F214, Y216, P217, P219, A220, E223
						Hbonds: I162, S202		Hbonds: C215

P-DNA = HP1043–DNA interface; P–P = HP1043 dimer cleft (A chain–B chain); Bi = B chain domain interface; A ext = external surface of the A chain; B ext = external surface of the B chain; contacts = hydrophobic contacts; Hbonds = hydrogen bonds; in bold: experimentally tested molecules.

**FIGURE 4:**
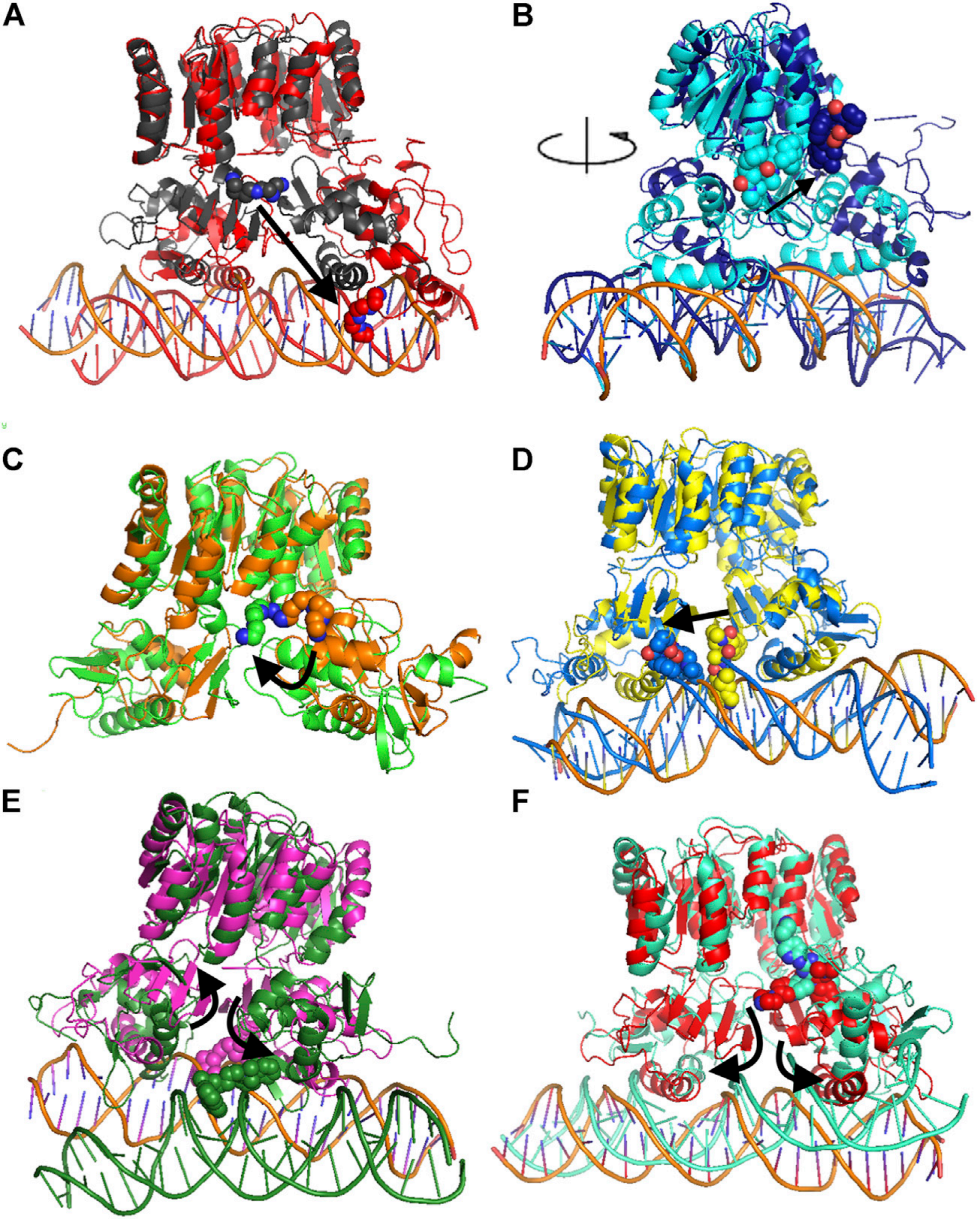
Comparison of MD representative conformation and docking pose. HP1043_DNA–tetraethylenepentamine **(A)**, HP1043_DNA–dihydroergotoxine **(B)**, HP1043–tetraethylenepentamine **(C)**, HP1043_DNA–ergotamine **(D)**, HP1043_DNA–hexafluronium **(E)**, and HP1043_DNA–ribociclib **(F)**. Ligand and domain movements are evidenced by black arrows. Docking conformation are in gray, light blue, orange, yellow, pink, and light red; representative MD conformation are colored as DNA in red, dark blue, green, blue, dark green, and aquamarine.

Molecular dynamics stability was evaluated based on backbone RMSD, and all complexes reached equilibrium during the simulation (Supplementary Figure S1). Two complexes, namely, HP1043_DNA-tetraethylenepentamine and HP1043_DNA-dihydroergotoxine, displayed the expulsion of the ligand during the simulation (Figures 4A,B), suggesting a lower affinity of these drugs for the HP1043_DNA complex, despite the negative binding energy between the drug and HP1043.

Considering ligand binding, as confirmed by interacting residue analysis, most complexes maintained a stable position (see below) except for HP1043_DNA–ponatinib, HP1043_DNA–osimertinib, and HP1043_DNA–tubocurarine complexes, where the three drugs showed a higher affinity for the DNA molecule than the transcription factor. Thus, they maintained only interactions with DNA, suggesting a low specificity of these drugs for HP1043. In addition, ligand tetraethylenepentamine bound to the A chain of HP1043 moved to the P–P interface during the simulation, and this result superimposed to the binding position of the complex with tetraethylenepentamine bound to the P–P interface of HP1043 (Figure 4C). Finally, ligand ergotamine, bound to HP1043–DNA, moved toward the A chain, reducing the interaction interface between protein and DNA and causing the displacement of helix H8, necessary for DNA interaction, a partial unfolding of C-terminal domain of the A chain and increasing the DNA bending (Figure 4D).

**FIGURE 5:**
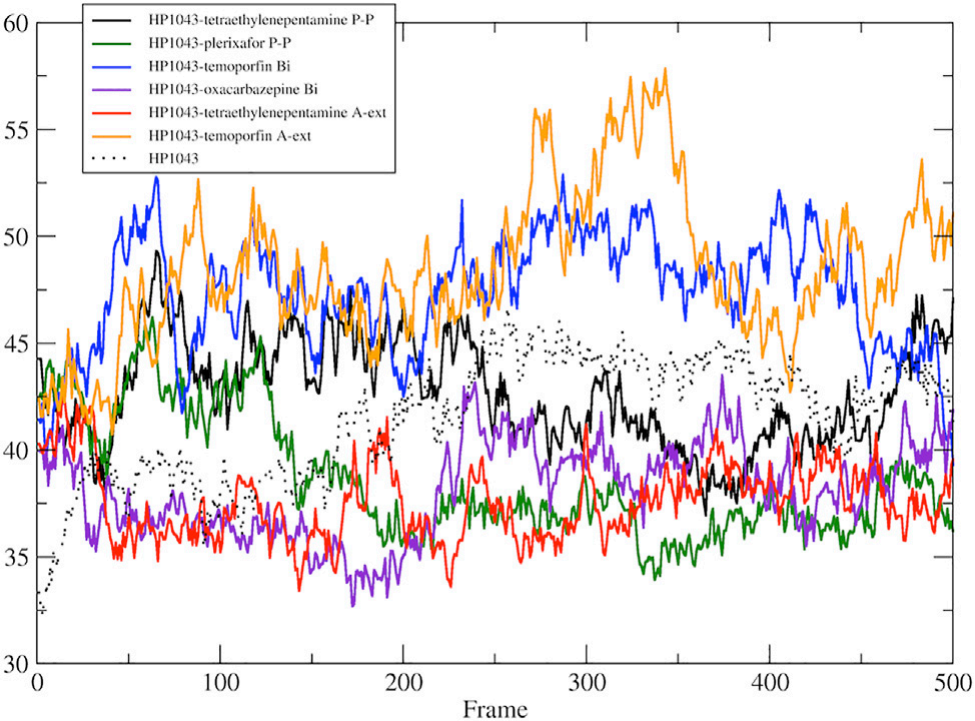
DNA-binding domain distance in the free form. Distance between the center of mass of DNA-binding domain of A chain and B chain.

A subset of tested drugs induced movements of the C-terminal domains. Complexes bound to hexafluronium and ribociclib displayed a mutual rotation of the C-terminal domains that increased its distance (Figures 4E,F), while plerixafor, oxcarbazepine, and both complexes with tetraethylenepentamine bound to HP1043, without DNA, displayed a reduction of domain distance (Figure 5). The remaining complexes maintained the conformation of the C-terminal domain during the whole simulation.

Considering the residue fluctuation of complexes without DNA, residues belonging to the DNA-binding domain displayed major flexibility (Supplementary Figure S2). On the other hand, all complexes presented reduced flexibility of A chain residues 120–145 compared to HP1043 unbound to ligands (red line in Supplementary Figure S2). These residues belonged to the DNA-binding domain and localized at the domain interface with the dimerization domain. Residues 170–190, belonging to the A chain and composing the interface to DNA (green line in Supplementary Figure S2), also displayed a reduced flexibility for all bound complexes except for both complexes bound to tetraethylenepentamine. Overall, HP1043–tetraethylenepentamine_Aext complex displayed reduced flexibility all along the structure, compared to the other complexes. Analyzing HP1043 complexes bound to DNA, the residue flexibility was generally increased compared to the unbound complex. Above all, HP1043_DNA bound to the dihydroergotoxine molecule, as previously mentioned, displayed the unbinding of C-terminal domain from the DNA molecule. Differences in local flexibility can be evidenced for residues 188–200 (A chain) (yellow line in Supplementary Figure S2), matching to helix H8 involved in DNA binding, belonging to complex HP1043_DNA bound to hexafluronium, ergotamine, and tetraethylenepentamine. Residues 389–390 (166–168 B chain) corresponding to the domain interface loop (blue arrow in Supplementary Figure S2), showed higher flexibility in complexes bound to dihydroergotoxine, hexafluronium, ponatinib, and plerixafor molecules. Finally, B chain helix H8 (gray line in Supplementary Figure S2) displayed increased flexibility in complexes HP1043_DNA–dihydroergotoxine, HP1043_DNA–hexafluronium, and HP1043_DNA–ponatinib.

#### 3.1.3 Drug Binding

The MMGBSA approach was applied to estimate the binding energy of drug ligands to the HP1043 transcription factor with results reported in Table 2. Binding free energy values ranged between −6.65 and −84.34 kcal/mol. Ligands bound at the protein–DNA interface (molecules in P-DNA–binding site in Table 2) displayed a lower affinity (−35.27 to −10.56 kcal/mol) compared to drugs localized at the P–P binding site (−75.23 to −24.23), due to fewer contacts/hydrogen bonds (listed in “MD interaction residues” of Table 2). Moreover, these drugs principally bound the DNA molecule, showing a low specificity for the HP1043 transcription factor. Considering the drugs bound only to the transcription factor, two left the complex during the simulation (tetraethylenepentamine and dihydroergotoxine), and thus, their binding energy estimation cannot be considered a reliable value. Among the others, plerixafor and trientine showed estimated binding energy of −75.23 and −44.38 kcal/mol, respectively. Drugs evaluated on the HP1043 free complex presented similar conditions, and molecules bound to a single domain or on the external surface displayed lower affinity, except for tetraethylenepentamine. Instead, drugs bound at the dimer cleft (plerixafor and tetraethylenepentamine) showed an estimated binding energy of −84.34 and −58.09 kcal/mol, respectively, thus, resulting in a higher affinity for HP1043.

The binding cavities identified during docking simulations were partially maintained. From the interacting residue analysis, three main residue groups were identified: Tyr360 (Tyr137 B), Glu364 (Glu141 B), Val365 (Val142 B), Phe372 (Phe149 B), and Thr376 (Thr153 B) at the B chain—DNA interface (red in Figure 3 and double-underlined in Table 2); Glu133, Lys145, Glu174, Glu175, and Glu355 (Glu132 B) at the dimer cleft (P–P) involving the residues of DNA-binding domain of both chains (green in Figure 3 and single underlined in Table 2); and Ala111, Glu311 (Glu88 B) at the dimerization domain interface (yellow in Figure 3 and dotted-underlined in Table 2). All the listed residues are involved both in hydrophobic contacts and hydrogen bonds with analyzed drugs (see “MD interaction residues” in Table 2 for details).

#### 3.1.4 DNA Binding

**FIGURE 6:**
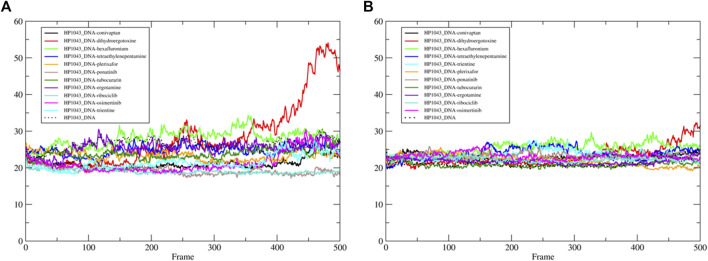
HP1043–DNA distance. Distance between A chain and DNA center of mass **(A)** and between B chain and DNA center of mass **(B)**.

We estimated the binding energy between the DNA molecule and HP1043 transcription factor bound to tested drugs with values reported in Table 3. All complexes displayed remarkable negative binding energy, except for HP1043_DNA bound to hexafluronium ligand, and this value corresponds to an increased distance between the A chain, or B chain, and the DNA molecule, evidenced in Figure 6. Also, complex HP1043_DNA–dihydroergotoxine showed no binding of the A chain to DNA (Figure 6). A distance increment between the B chain and the DNA molecule, but for partial simulation time, was also displayed by HP1043_DNA–dihydroergotoxine and HP1043_DNA–trientine complexes, which presented a reduced affinity compared to the other complexes for DNA.

**TABLE 3:** HP1043-DNA-binding energy estimation (MMGBSA); distance of DNA key residues compared to HP1043_DNA.

	Binding site	Molecule name	MMGBSA P-DNA (kcal/mol) (SD)	Helix α8						β11–β12		Helix α8						β11–β12	
				I188	N189	R192	Q193	D196	K197	T206	R208	I411	N412	R415	Q416	D419	K420	T429	R431
HP1043-DNA	P-DNA	hexafluronium	−9.95 (±21.07)	+	+	+	+	+	+		−	+	+	+	+	+	+	+	+
		ponatinib	−62.08 (±20.49)	−	−	−	−	−	−					+	+	+	+		
		osimertinib	−52.86 (±20.07)								+			+		+	+		
		tubocurarin	−63.51 (±16.27)																
		ergotamine	−62.99 (±19.32)	+	+	+	+	+	+										
		conivaptan	−57.33 (±20.8)				−	−	−										
	P–P	plerixafor	−47.72 (±21.7)					−	−		−								
		tetraethylenepentamine	−56.29 (±17.28)					+	+					+	+	+	+		
		trientine	−27.47 (±26.67)									+	+	+	+		+		
		ribociclib	−81.81 (±17.17)	−	−	−	−	−	−							−	−		
	B ext	dihydroergotoxine	−30.64 (±20.54)	+	+	+	+	+	+	+	+	+	+	+	+	+			

The distances between key residues for DNA binding (Zannoni et al., 2021) and DNA molecules were observed during trajectories, whose results are reported in Table 3. These data confirmed no DNA binding in complexes HP1043_DNA–hexafluronium and HP1043_DNA–dihydroergotoxine since both chains showed a distance increase, and the displacement of the A chain in complex HP1043_DNA–ergotamine, and of the B chain in complex HP1043_DNA–trientine.

### 3.3 *In Vitro* Inhibition of HP1043 DNA-Binding Activity

**FIGURE 7:**
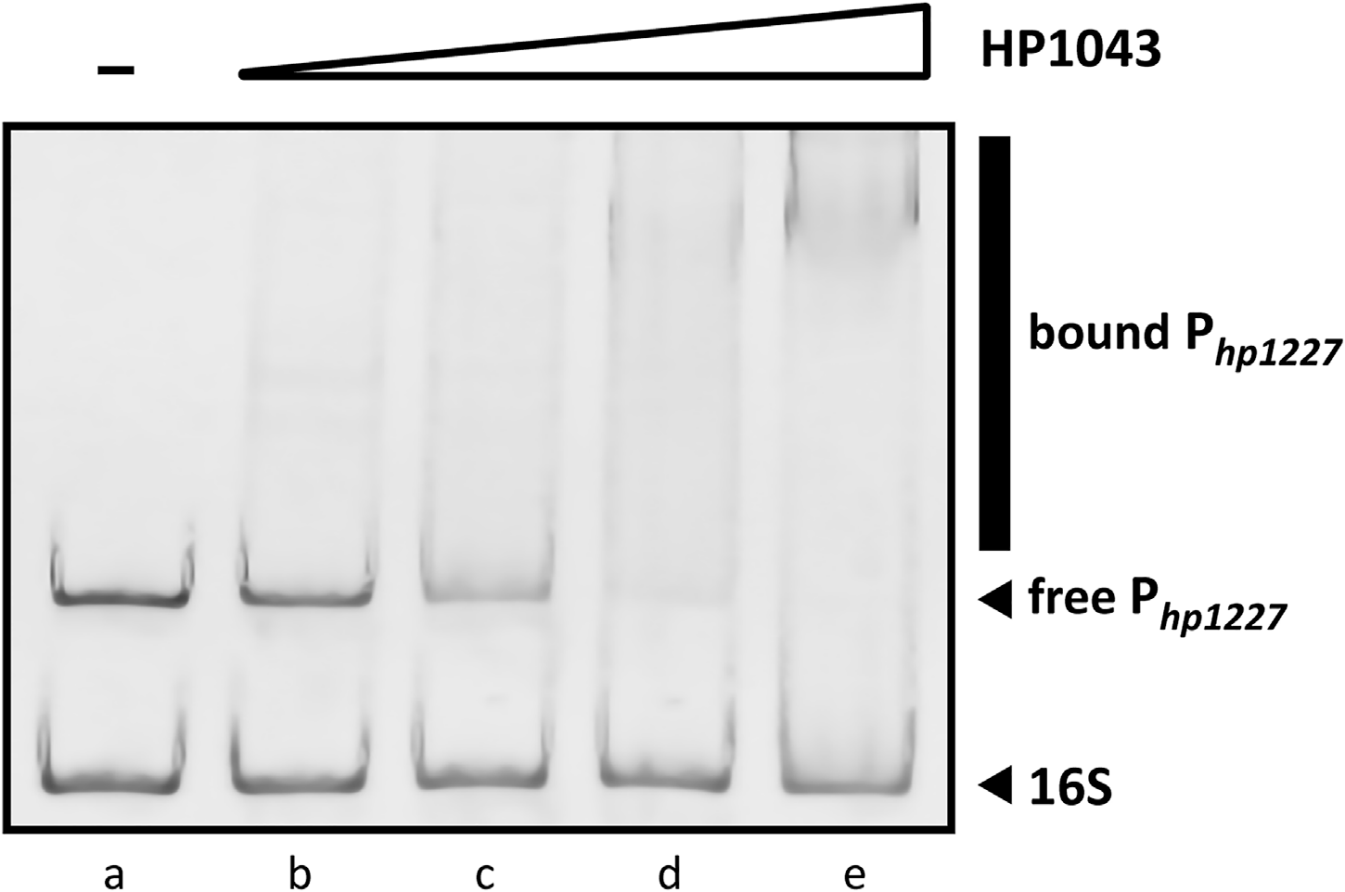
Titration of the 190-bp specific DNA probe with HP1043. All samples contained two DNA probes, one specific (P*_hp1227_*) and another non-specific (16S rRNA gene). For each reaction, 10 ng of each DNA probe was used. Lane (a) control reaction without HP1043 protein; lane (b) to (e) samples containing, respectively, 1, 2, 4, and 8 μM of HP1043 protein. DNA probes were mixed with increasing concentrations of the recombinant protein, incubated at room temperature for 30 min, and subjected to a 6% PAGE. EMSA analysis show a decrease in free probe (P*_hp1227_*) in response to increasing amounts of HP1043 protein, indicative of the formation of specific protein–DNA complexes represented by a smear (marked with a vertical line on the right side of the image). The smear represents protein–DNA complexes dissociating during electrophoresis.

Previous studies have documented the consistent ability of HP1043 to bind specific sequences embedded in several promoters, proving its crucial role in the viability of *H. pylori* (Pelliciari et al., 2017; Zannoni et al., 2021). The EMSA is a versatile and sensitive tool for detecting protein–nucleic acid interaction and its inibition. To determine *in vitro* whether the recombinant HP1043 protein retained its biological activity to bind the target promoter P_*hp1227*_, we evaluated the shift in electrophoretic mobility of the DNA probe after protein binding on a polyacrylamide gel (see Materials and Methods Section 2.4). EMSA analyses showed a decrease of the unbound DNA in a concentration-dependent manner (Figure 7). We chose a concentration of 4 µM HP1043 monomer for subsequent tests on putative selected inhibitors, a non-completely saturating binding condition. Moreover, a 127-bp probe from the 16S gene was used as a non-specific probe for HP1043 binding in each reaction.

Among ligands previously selected by molecular dockings and dynamics, those bound to DNA molecules were excluded to prevent non-specific interactions. The seven ligands available for sale (plerixafor, tetraethylenepentamine, trientine, ribociclib, dihydroergotoxine, oxcarbazepine, and temoporfin) were tested to evaluate inhibition properties through EMSA. In particular, the recombinant HP1043 protein was incubated with the validated target P_*hp1227*_ in the presence of decreasing concentrations of ligand (from 1 mM to 50 µM). DNA-binding inhibition could indicate whether dynamic interactions between the compound and HP1043 hinder the formation of the protein-promoter complex. Since the compounds were diluted in either H_2_O or DMSO, negative control reactions were included in EMSA analysis, where equivalent volumes of solvent were added to the protein–DNA mixes replacing the ligands. In addition, we prepared a second set of negative controls to remove the hypothesis that the ligand itself might induce a mobility shift of DNA probes. For these reactions, 1 × binding buffer was used instead of the HP1043 dilution. The magnitude of the inhibitory effect was deduced from the optical density of the free DNA bands, and the ligands were considered inhibitors when capable of interfering with the protein-dependent specific shift of P_*hp1227*_.

**FIGURE 8:**
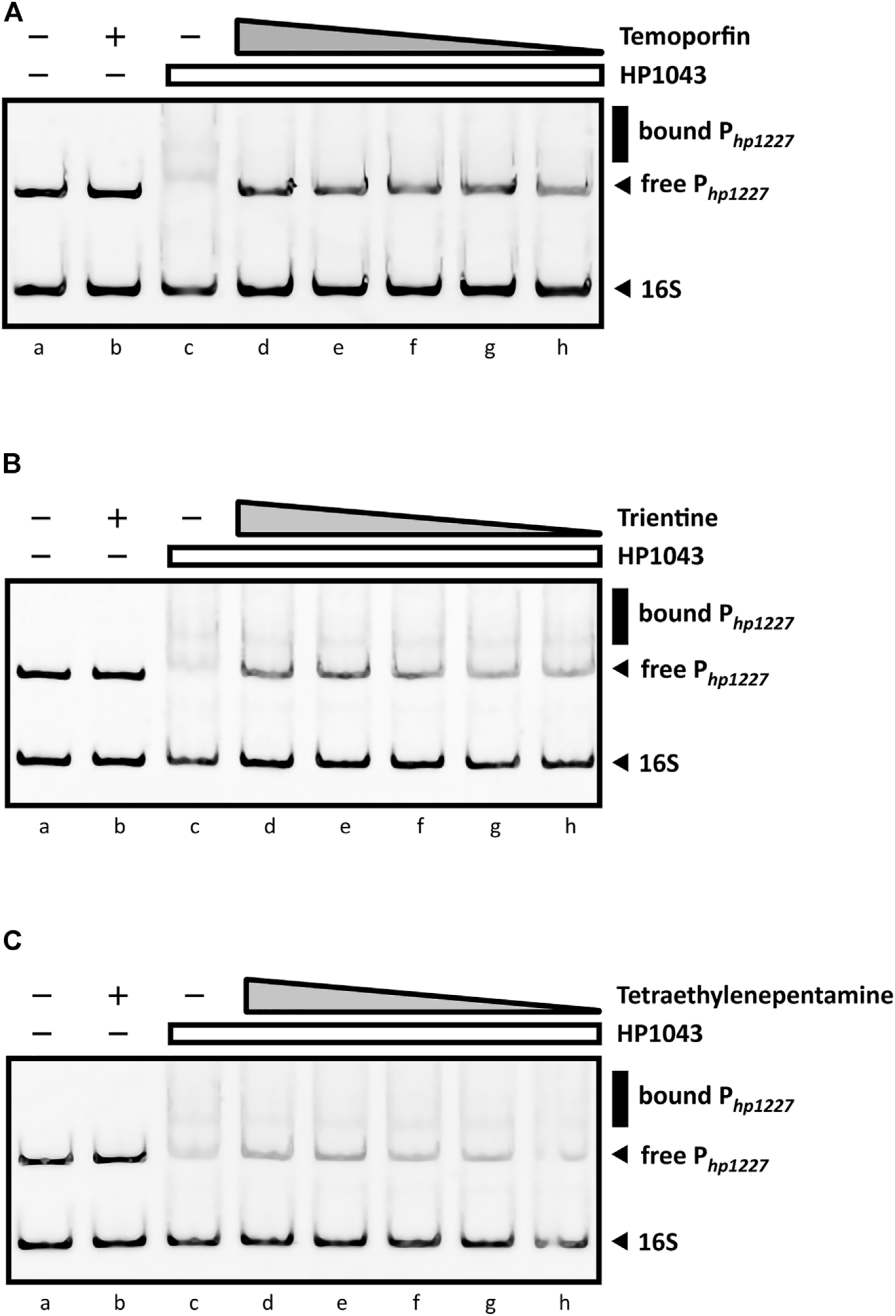
EMSAs in the presence of DNA-binding inhibitors. All EMSAs show the same amount of specific (P*_hp1227_*) and non-specific (16S rRNA gene) DNA probes and the same sample order: lane (a) DNA probes control, protein- and ligand-free; (b) compound control at 1 mM (indicated by a “+”) without the protein; (c) DNA-binding control in the presence of HP1043; lane (d) to (h) show samples containing a fixed amount of HP1043 monomeric protein (4 μM) with a decreasing concentration of the ligand, respectively, 1, 0.5, 0.2, 0.1, and 0.05 mM; the absence of protein and compound is indicated by a “−”; the compound concentration is depicted as a gray triangle, while a white rectangle is used for the HP1043 protein fixed concentration. **(A)** Addition of temoporfin results in a reduced *in vitro* affinity of HP1043 protein for its target promoter region. Temoporfin restored the free DNA probe mobility and reduced the smear of the specific DNA probe at higher concentrations. For **(B)** trientine and **(C)** tetraethylenepentamine, similar inhibition effects were observed. Both chemicals induced a decreased DNA-binding activity for HP1043. However, a faint up-shifted band is still visible with the tested conditions. Symbols are as described in the legend in Figure 7.

As shown in Figure 8A, the sharp inhibitory effect for temoporfin was detected even at 50 µM (lane h, Figure 8A). Such concentrations correspond to a mole monomeric-HP1043:ligand ratio of 1:12. At higher concentrations (lane d, e, f, and g; Figure 8A), reaching a mole ratio of 1:250, temoporfin significantly reduced the mobility shift of the specific DNA probe. Also, trientine (Figure 8B) and tetraethylenepentamine (Figure 8C) exhibit a less marked but appreciable loss of DNA-binding activity of HP1043. However, a faint band corresponding to the bound promoter DNA was still detected in both EMSA. Regardless, all three ligands were able to restore the electrophoretic mobility of free P_*hp1227*_ in a concentration-dependent manner. In contrast, four ligands did not show an appreciable reduction of the DNA–protein complex under the used experimental conditions (Supplementary Figure S3). In conclusion, three candidates (temoporfin, tetraethylenepentamine and trientine) showed a sharp inhibition pattern on HP1043 binding, thus suggesting that these drugs can bind with a high affinity to HP1043.

## 4 Discussion

*H. pylori* colonizes the gastric mucosa of about 50% of the human population. It is strongly associated with the inflammation of the upper gastrointestinal tract, and it is related to several diseases including gastric cancer. Currently, the treatment for eradication of *H. pylori* infection mainly consists of triple standard therapy, including a proton pump inhibitor, amoxicillin and clarithromycin (Zagari et al., 2018; Roszczenko-Jasińska et al., 2020), frequently supplemented with bismuth salt, and substituted by tetracycline and metronidazole when required. The main issue of this therapy is the antibiotic resistance to clarithromycin and metronidazole, affecting the treatment efficacy in about 70% of the cases (Su Young Kim et al., 2015; Hooi et al., 2017; Roszczenko-Jasińska et al., 2020). Known antibiotics target different bacterial enzymes and ribosomal subunits (Hu et al., 2017), but none target a transcription factor. Recently, several novel molecules with an anti-*H. pylori* effect were proposed (Nishimori et al., 2007; Geng et al., 2009; Makobongo et al., 2014), also targeting the HP1043 transcription factor (González et al., 2019b; 2019a). The discovery of new therapies against *H. pylori* requires the identification and validation of novel drug targets essential for *in vivo* growth or pathogenicity (González et al., 2018; Roszczenko-Jasińska et al., 2020). Transcription factors belong to the genes essential for the growth and virulence of pathogenic bacteria, as they act on more than one target gene. In *H. pylori*, one of these is HP1043 (Pelliciari et al., 2017). This allows us to consider HP1043 as a stable protein, as it is subjected to an evolutionary pressure that avoids the emergence of new mutants.

The development of new drugs is a time-consuming and expensive process with a high failure risk. Drug repositioning has become a strategy to reduce time and costs, proposing new applications for known drugs. This strategy was recently applied to identify treatments for several diseases such as tumors (Aydin et al., 2022; Persico et al., 2022; Petrosyan et al., 2022), cardiac diseases (Rouhana et al., 2021), and neurodegenerative diseases (Bhat et al., 2020; Agostini et al., 2021).

**FIGURE 9:**
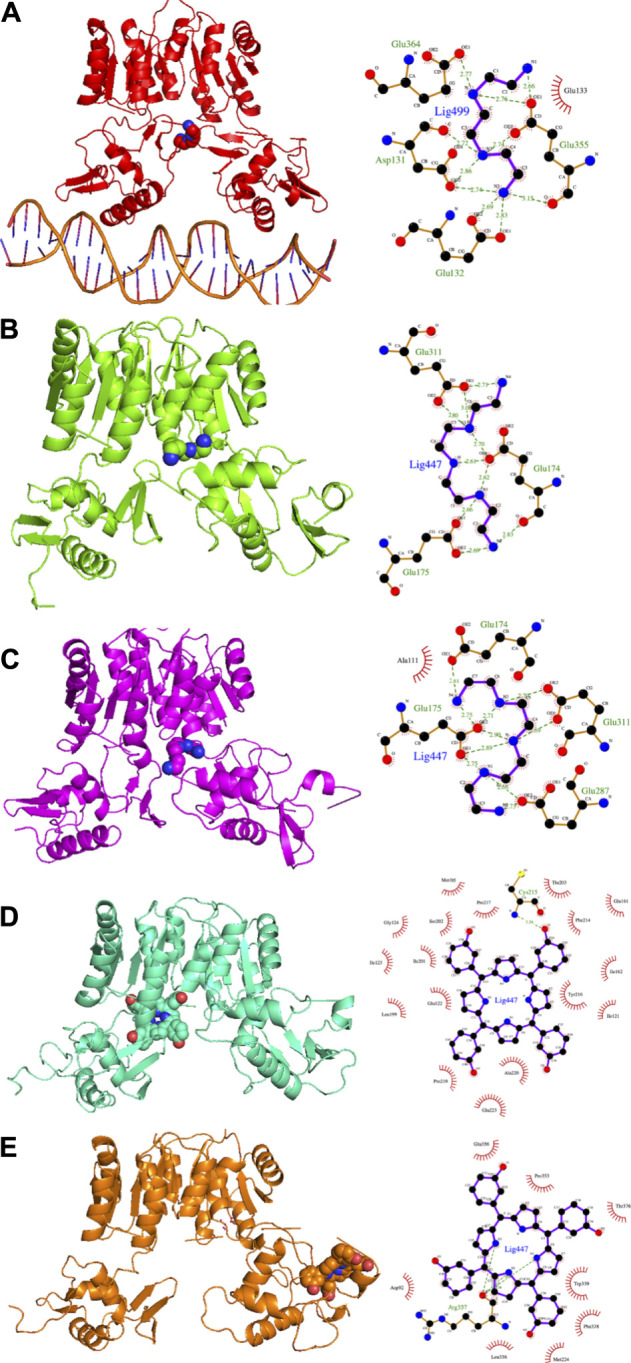
Candidate drugs binding mode obtained from MD and interaction analysis. MD representative conformation of HP1043_DNA–trientine **(A)**, HP1043–tetraethylenpentamine_Aext **(B)** and P–P **(C)**, HP1043_termoporfin Aext **(D)** and Bi **(E)** and related Ligplot sketch of interaction analysis; dashed lines represent H-bonds and red eyelashes identify residues involved in hydrophobic contacts.

In the present study, we propose a repositioning of approved drugs for HP1043 by applying the VHTS protocol to select molecules with a high affinity for this protein both in the free form and bound to the DNA. The binding mode of top results was deeply evaluated by molecular dynamics and experimentally tested for inhibition properties through EMSA. Presented results evidenced three promising drugs (binding mode obtained from molecular dynamics and interaction analysis are reported in Figure 9), displaying an appreciable impairment of DNA-binding activity of HP1043, excluding non-specific binding of DNA. Trientine was *in silico* analyzed as bound to the HP1043–DNA complex, and displayed a reduced affinity for DNA molecule, compared to the other tested complexes, just as an increased distance between the B chain DNA–binding domain and the DNA molecule. The other two drugs, namely, tetraethylenepentamine and temoporfin, were simulated and bound to HP1043 in two different conformations. The first one showed high affinity for the transcription factor without evidence on protein flexibility or conformation, while the latter induced the increase of chain distance, inducing conformational changes on the protein structure.

From the pharmacological viewpoint, trientine is a Cu chelating agent used in the treatment of Wilson’s disease, and it is orally delivered and poorly absorbed from the gastrointestinal tract. Tetraethylenepentamine is an ethyleneamine with metal-chelating properties, while temoporfin is a photosensitizer used in photodynamic therapy of tumor cells; it is intravenously administered and collected in tumor tissues. These molecules belong to different pharmaceutical categories, but all display a similar inhibitory activity on HP1043 DNA binding.

Starting from the presented results, these three compounds can be considered to propose new molecules for *H. pylori* treatment, after having tested directly on bacteria to assess experimental Ki, as well as assays to determine the minimal inhibitory concentration (MIC) and the minimal bactericidal concentration (MBC) must be performed.

## Data Availability

The original contributions presented in the study are included in the article/Supplementary Material, further inquiries can be directed to the corresponding author.
