# Supplementary material for: Targeting the Essential Transcription Factor HP1043 of Helicobacter pylori: A Drug Repositioning Study
Source: Front Mol Biosci. 2022 May 11;9:887564. doi: 10.3389/fmolb.2022.887564 (PMC9135449; doi:10.3389/fmolb.2022.887564)
Supplement: Supplementary file 1 [file DataSheet1.pdf]

Supplementary Material

Targeting the essential transcription factor HP1043 of *Helicobacter pylori*: a drug repositioning study

Federico Antoniciello[1], Davide Roncarati[1], Annamaria Zannoni[1,#], Elena Chiti[1], Vincenzo Scarlato[1], Federica Chiappori[2*]

1 Supplementary Tables

1.1 Table S1 Plasmids used in this study

Plasmid	Description	Source/Reference
pTrc::1043	Derivative of pTrcHisA expressing the HP1043 response regulator; Amp^r	(Delany et al., 2002)
pGEMt-*P*1227 WT	pGEMt-Easy derivative, containing a 61 bp probe corresponding to the region from 1.290.664 to 1.290.725 of *H. pylori* G27 genome. The region corresponds to the promoter of *HPG27_RS06145* gene (*hp1227* according to *H. pylori* 26695 strain annotation)	(Pelliciari et al., 2017)

1.2 Table S2 Oligonucleotides used in this study

Primer	Sequence	Source/Reference
**Php1227_EMSA_F**	GCCAAAACGCCTAAAGCC	(Zannoni et al., 2021)
**Php1227_EMSA_R**	TTGAAAGCGCAATAACCGC	(Zannoni et al., 2021)
**16SRTF**	GGAGTACGGTCGCAAGATTAAA	(Pelliciari et al., 2017)
**16SRTR**	CTAGCGGATTCTCTCAATGTCAA	(Pelliciari et al., 2017)

1.3 Table S3 Docking comparison to literature

Docking box have dimension limits, so to include the whole structure, two docking boxes were designed, HP1043_site1 and Hp1043_site 2. Boxes overlap at dimer interface.

		**HP1043-DNA**			**HP1043_site1**			**HP1043_site2**		
		**binding energy**	**# cluster**	**Ki**	**binding energy**	**# cluster**	**Ki**	**binding energy**	**# cluster**	**Ki**
nifedipine	ZINC85205448	-6.15	25	31.28 uM	-8.25	9	899.53 nM	-6.17	14	29.83 uM
nicardipine	ZINC19796087	-8.94	10	277.87 nM	-8.70	13	419.39 nM	-6.69	13	12.41 uM
	ZINC84400879	-8.39	10	707.68 nM	-7.10	2	6.29 uM	-7.81	12	1.90 uM
nisoldipine	ZINC591993	-5.53	13	88.97 uM	-6.80	6	10.36 uM	-6.84	22	9.70 uM
	ZINC19632706	-8.60	4	497.04 nM	-7.19	12	5.33 uM	-7.27	21	4.67 uM
nimodipine	ZINC19632713	-5.80	16	55.67 uM	-6.35	28	22.10 uM	-7.08	21	6.41 uM
	ZINC19632718	-5.03	11	205.41 uM	-5.83	11	53.39 uM	-8.05	8	1.26 uM
nitrendipine	ZINC84403367	-5.72	24	63.63 uM	-7.36	26	4.03 uM	-6.53	39	16.44 uM
	ZINC100001908	-7.70	10	2.29 uM	-7.69	93	2.32 uM	-7.62	46	2.58 uM
lercandipine	ZINC19685788	-8.04	7	1.27 uM	-7.34	4	4.15 uM	-7.82	1	1.85 uM
	ZINC19685790	-10.07	1	41.87 nM	-9.02	1	243.13 nM	-8.84	1	333.09 nM
chrysin	ZINC3872070	-8.80	83	353.26 nM	-8.70	34	416.11 nM	-9.14	9	201.10 nM
apigenin	ZINC3871576	-9.16	65	192.69 nM	-8.35	12	762.31 nM	-8.21	11	960.09 nM
luteolin	ZINC18185774	-8.73	88	397.61 nM	-10.10	5	39.33 nM	-8.84	17	331.86 nM
hesperetin (yso2)	ZINC39092	-8.98	92	260.60 nM	-7.72	72	2.19 uM	-7.56	89	2.86 uM
kaempferol	ZINC3869768	-8.46	16	627.90 nM	-7.86	10	1.72 uM	-7.70	47	2.28 uM
quercitin (meletin)	ZINC3869685	-8.77	34	371.41 nM	-8.09	10	1.17 uM	-9.44	9	121.28 nM
myricetin	ZINC3874317	-9.64	42	85.18 nM	-7.41	24	3.71 uM	-8.15	46	1.06 uM

1.4 Table S4 docking results

ligand	binding energy (kcal/mol)	run	Ki	
**ZINC000022443609_HP1043-DNA**	**-23.84**	**38**	**3.37 aM**	
ZINC000013492579_HP1043-DNA	-16.79	117	494.39 fM	Not for sale
ZINC000003938681_HP1043-DNA	-16.41	66	937.24 fM	DNA
**ZINC000019363537_HP1043-DNA**	**-15.57**	**81**	**3.86 pM**	
**ZINC000001566899_HP1043-DNA**	**-15.41**	**98**	**5.06 pM**	
ZINC000004214772_HP1043-DNA	-15.31	147	5.96 pM	DNA
ZINC000100088802_HP1043-DNA	-14.99	156	10.26 pM	DNA
ZINC000150338708_HP1043-DNA	-14.39	11	28.4 pM	Expensive
ZINC000013492553_HP1043-DNA	-14.3	190	33.17 pM	Not for sale
ZINC000028823338_HP1043-DNA	-14.14	124	43.44 pM	Not for sale
ZINC000004097448_HP1043-DNA	-14.05	43	50.02 pM	DNA
ZINC000004097404_HP1043-DNA	-13.99	79	55.55 pM	DNA
ZINC000222508879_HP1043-DNA	-13.99	75	55.58 pM	Not for sale
ZINC000028823530_HP1043-DNA	-13.93	60	61.88 pM	Not for sale
ZINC000013494070_HP1043-DNA	-13.92	42	63.08 pM	Not for sale
**ZINC000019364225_HP1043-DNA**	**-13.84**	**70**	**71.87 pM**	
ZINC000013494068_HP1043-DNA	-13.73	46	86.02 pM	Not for sale
ZINC000043206370_HP1043-DNA	-13.56	176	115.23 pM	DNA
ZINC000001612996_HP1043-DNA	-13.32	199	171.12 pM	DNA
ZINC000013494069_HP1043-DNA	-13.1	10	250.98 pM	Not for sale
**ZINC000036701290_HP1043-DNA**	**-13.08**	**103**	**256.35 pM**	
ZINC000028823491_HP1043-DNA	-12.92	139	339.8 pM	Not for sale
ZINC000087666889_HP1043-DNA	-12.92	22	335.64 pM	Not for sale
**ZINC000098023177_HP1043-DNA**	**-12.89**	**92**	**356.1 pM**	
ZINC000150598297_HP1043-DNA	-12.82	121	401.34 pM	Not for sale
ZINC000004097383_HP1043-DNA	-12.81	40	406.52 pM	
ZINC000028134101_HP1043-DNA	-12.79	153	421.91 pM	Not for sale
ZINC000028823512_HP1043-DNA	-12.79	70	420.99 pM	Not for sale
ZINC000087666882_HP1043-DNA	-12.79	140	420.91 pM	Not for sale
**ZINC000072316335_HP1043-DNA**	**-12.7**	**128**	**487.81 pM**	
ZINC000022448983_HP1043-DNA	-12.66	167	526.94 pM	
ZINC000028823490_HP1043-DNA	-12.56	47	616.87 pM	Not for sale
ZINC000028823510_HP1043-DNA	-12.53	9	652.73 pM	Not for sale
**ZINC000003978083_HP1043-DNA**	**-12.46**	**143**	**737.86 pM**	
ZINC000030690433_HP1043-DNA	-12.46	46	740.65 pM	Expansive

**ZINC000052955754_HP1043-DNA**	**-12.46**	**31**	**736.06 pM**	
**ZINC000012503187_HP1043-DNA**	**-12.42**	**134**	**791.48 pM**	
**ZINC000014880002_HP1043-DNA**	**-12.39**	**15**	**827.22 pM**	
ZINC000087666886_HP1043-DNA	-12.38	137	837.75 pM	Not for sale
ZINC000222508821_HP1043-DNA	-12.32	63	936.79 pM	Not for sale
ZINC000028823531_HP1043-DNA	-12.29	149	985.83 pM	Not for sale
**ZINC000022443609_HP1043**	**-17.65**	**35**	**116.01 fM**	
**ZINC000019363537_HP1043**	**-13.68**	**21**	**94.13 pM**	
**ZINC000019363537_HP1043**	**-12.86**	**20**	**373.37 pM**	
**ZINC000003934128_HP1043**	**-12.23**	**146**	**1.09 nM**	
**ZINC000003934128_HP1043**	**-11.18**	**102**	**6.41 nM**	
ZINC000029059664_HP1043	-10.82	134	11.73 nM	Not for sale
ZINC000028824061_HP1043	-10.67	124	15.01 nM	Not for sale
ZINC000072126305_HP1043	-10.2	118	33.25 nM	Not for sale
ZINC000072126306_HP1043	-10.18	137	34.59 nM	Not for sale
ZINC000042804032_HP1043	-10.04	102	43.55 nM	Not for sale
**ZINC000000004724_HP1043**	**-9.94**	**103**	**51.39 nM**	
ZINC000044352037_HP1043	-9.91	178	54.83 nM	Not for sale
ZINC000029060547_HP1043	-9.7	127	76.97 nM	Not for sale
ZINC000029060386_HP1043	-9.55	108	99.38 nM	Not for sale

2 Reference

Delany, I., Spohn, G., Rappuoli, R., and Scarlato, V. (2002). Growth phase-dependent regulation of target gene promoters for binding of the essential orphan response regulator HP1043 of Helicobacter pylori. *J. Bacteriol.* 184. doi:10.1128/JB.184.17.4800-4810.2002.

Pelliciari, S., Pinatel, E., Vannini, A., Peano, C., Puccio, S., Bellis, G. De, et al. (2017). Insight into the essential role of the Helicobacter pylori HP1043 orphan response regulator: Genome-wide identification and characterization of the DNA-binding sites. *Sci. Rep.* 7. doi:10.1038/srep41063.

Zannoni, A., Pelliciari, S., Musiani, F., Chiappori, F., Roncarati, D., and Scarlato, V. (2021). Definition of the Binding Architecture to a Target Promoter of HP1043, the Essential Master Regulator of Helicobacter pylori. *Int. J. Mol. Sci. Artic.* doi:10.3390/ijms22157848.

3 Supplementary Figures

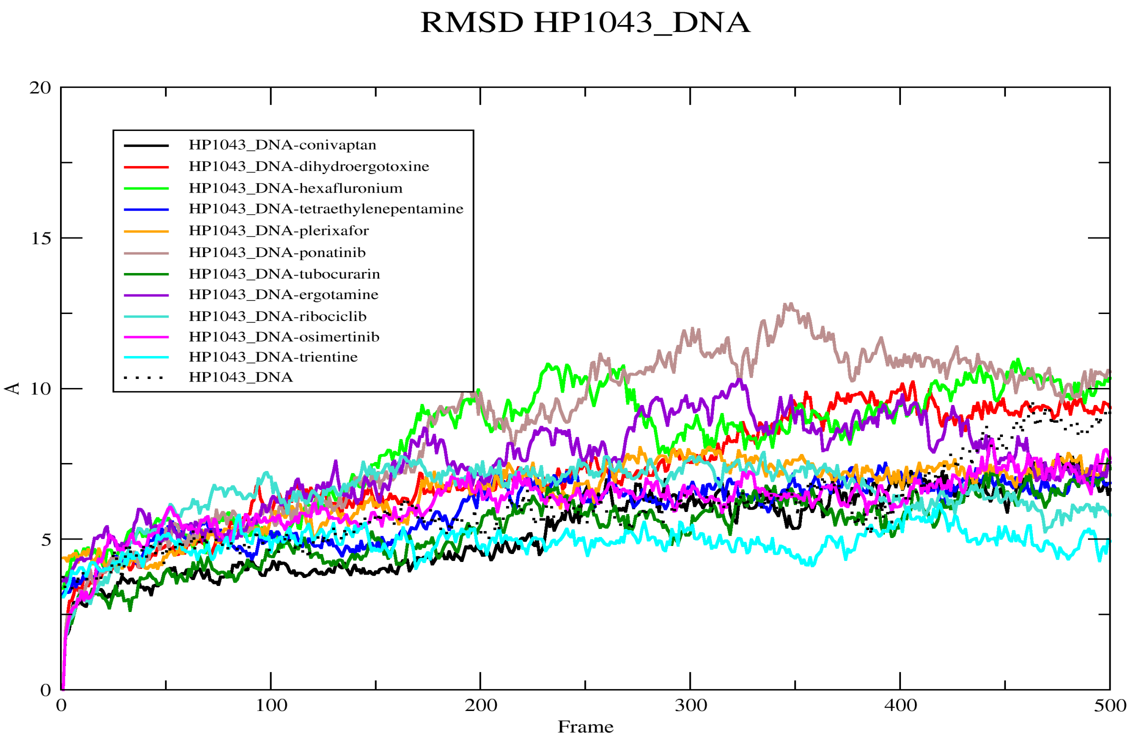

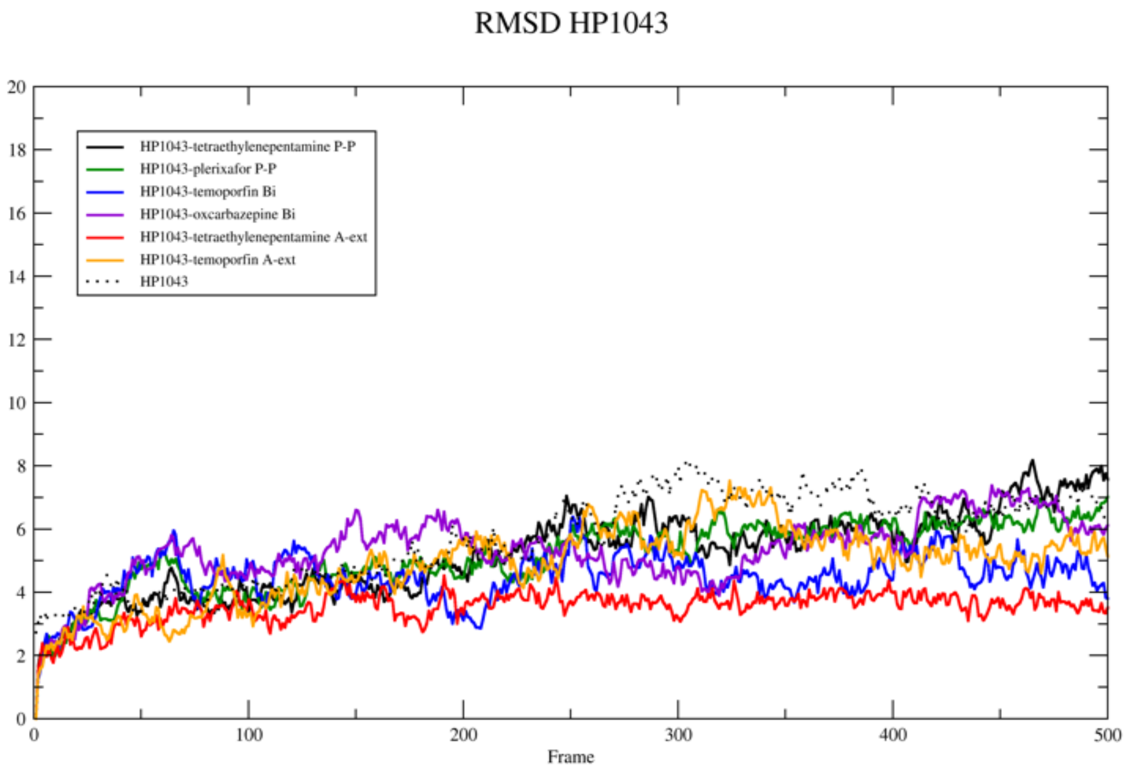

Supplementary Figure S1. Cα RMSD. On the top HP1043 bound to DNA complexes, bottom HP1043 free complexes.

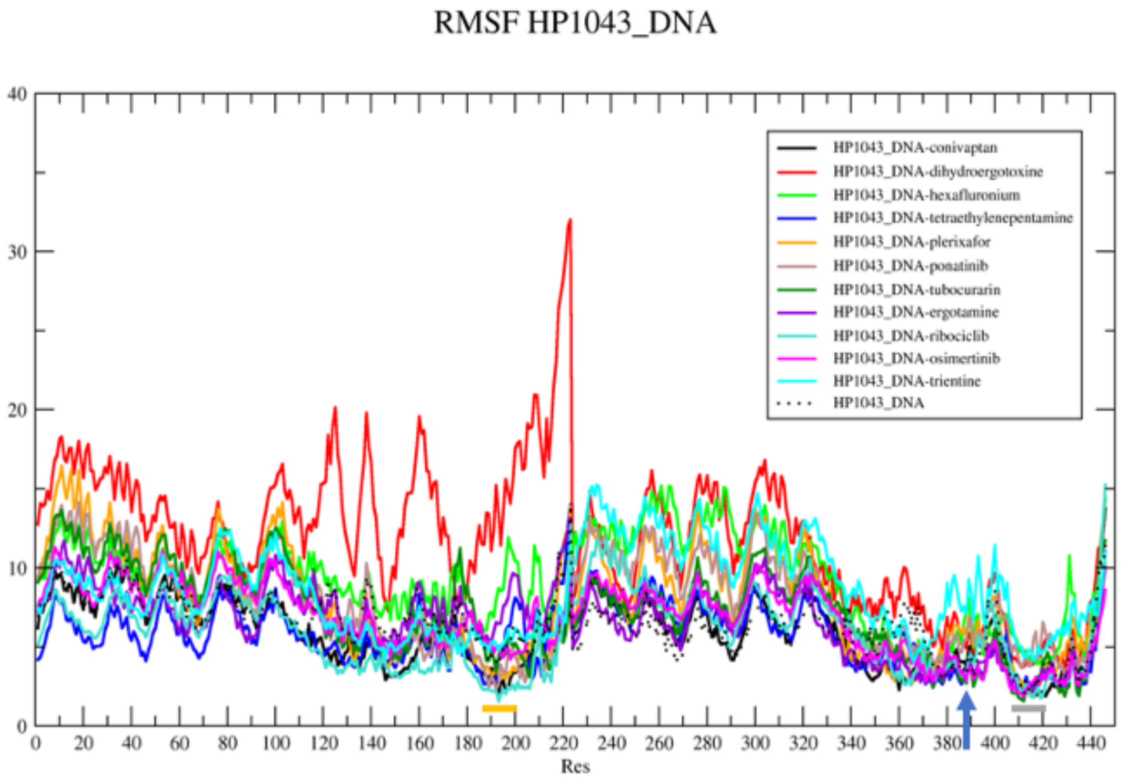

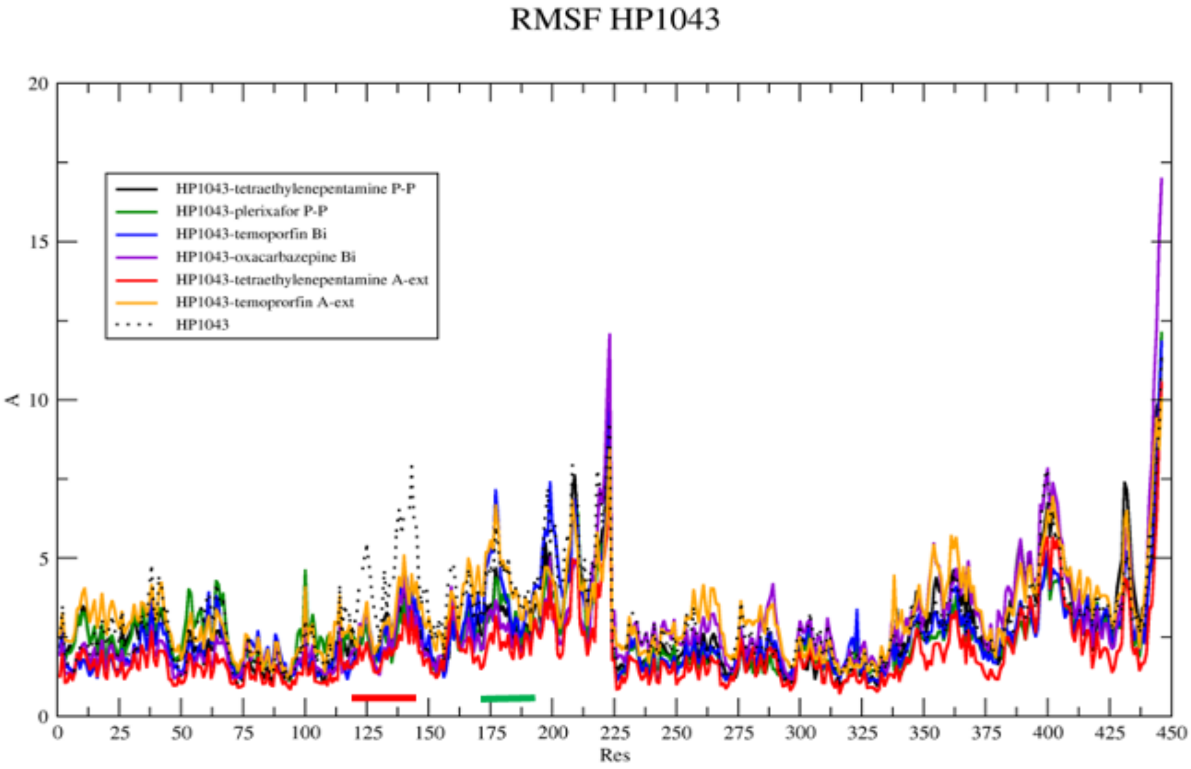

Supplementary Figure S2. RMSF per-residue. On the top HP1043 bound to DNA complexes, bottom HP1043 free complexes.

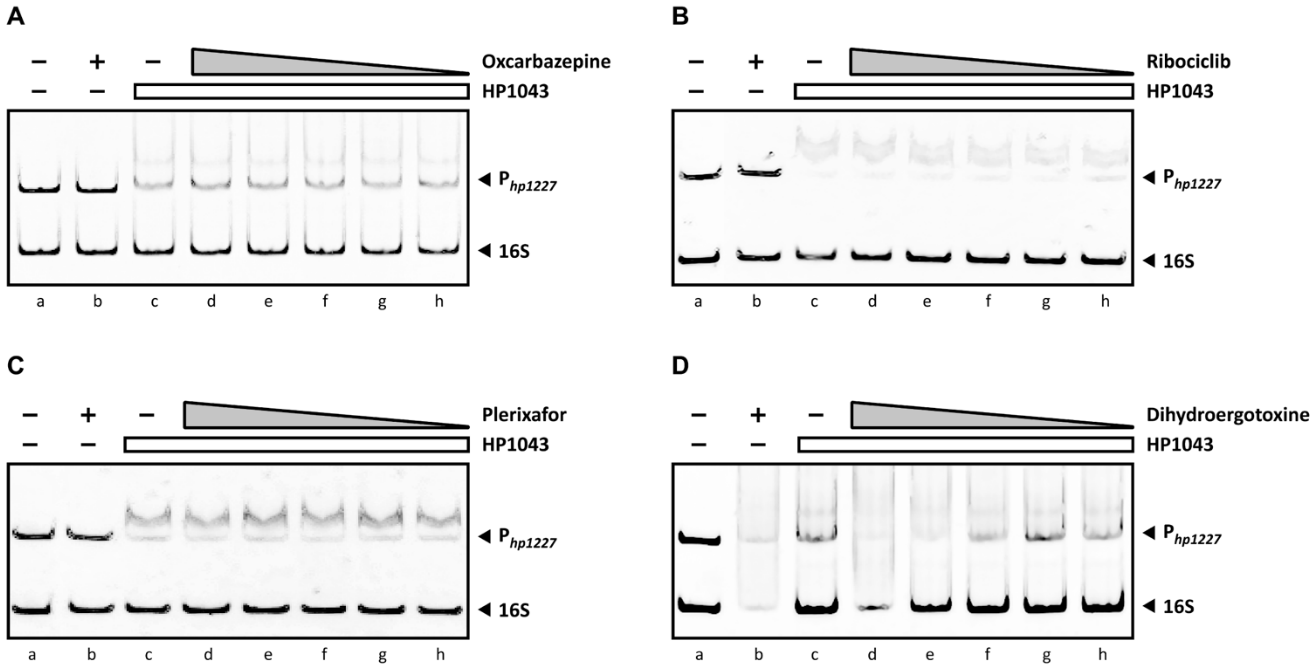

Supplementary Figure S3. EMSAs in the presence of putative ligands. All EMSAs show the same amount of specific (P_{hp1227}) and non-specific (16S rRNA gene) DNA probes and the same samples order: lane a) DNA probes control, protein- and ligand-free; b) compound control at 1 mM (indicated by a "+") without the protein; c) DNA binding control in the presence of HP1043; lane d) to h) show samples containing a fixed amount of HP1043 monomeric protein (4 µM) with a decreasing concentration of the ligand, respectively 1, 0.5, 0.2, 0.1, 0.05 mM; the absence of protein and compound is indicated by a "-"; the compound concentration is depicted as a grey triangle, while a white rectangle is used for the HP1043 protein fixed concentration. **A)** Oxcarbazepine did not cause a significant loss of the mobility shift if compared with the protein control (lane c). As well as **B)** Ribocilcib and **C)** Plerixafor, whose presence in the reaction mix did not affect the DNA binding. **D)** Dihydroergotamine interacted with both DNA probes at higher concentrations causing a shift in a protein-free reaction (lane b). Therefore, a putative inhibition was not assessable.
